# Acute beetroot juice supplementation augments early neuromuscular performance after blood flow restriction: a crossover study in elite basketball players

**DOI:** 10.3389/fnut.2025.1699766

**Published:** 2026-01-22

**Authors:** Runsheng Nie, Gengyini Zhang, Junhao Kong, Xi Wang, Zhiyu Xie, Lehai Lin, Tianhe Li, Yapu Liang

**Affiliations:** 1 School of Sport Science, Beijing Sport University, Beijing, China; 2 China Basketball College, Beijing Sport University, Beijing, China; 3 International Joint Laboratory on High Performance Sports Research, Beijing Sport University, Beijing, China; 4 School of Architecture and Urban Planning, Beijing University of Civil Engineering and Architecture, Beijing, China; 5 Department of Physical Education, Peking University, Beijing, China; 6 School of Strength and Conditioning Training, Beijing Sport University, Beijing, China; 7 Engineering Research Center of Ministry of Education for Key Core Technical Integration System and Equipment, Strength and Conditioning Training, Beijing Sport University, Beijing, China

**Keywords:** beetroot juice, blood flow restriction, post-activation potentiation, elite basketball players, jump performance

## Abstract

**Introduction:**

Beetroot juice (BJ), rich in nitrate, has been shown to enhance exercise performance via nitric oxide-mediated pathways. While its effects on endurance and strength have been studied, its influence on blood flow restriction (BFR)-induced post-activation potentiation (PAP) remains unclear.

**Methods:**

Twenty elite male basketball players (aged 21.25 ± 1.77 years) completed a randomized, double-blind, placebo-controlled crossover trial. Participants ingested BJ (8.4 mmol nitrate) or placebo before performing a standardized warm-up and BFR-assisted plyometric protocol. Counter-movement jump (CMJ) variables were recorded at baseline and at 0, 4-, 8-, 12-, and 16-min post-intervention. A two-way repeated-measures ANOVA was used to assess condition, time, and interaction effects, supplemented by Bonferroni-corrected paired *t*-tests and effect size analysis, with significance set at *p* ≤ 0.05.

**Results:**

BJ significantly increased jump height calculated from take-off velocity [H(v)], peak power (PP), and peak rate of force development (PRFD) during the first 8 min post-intervention compared to placebo. Placebo showed higher H(v) at 12 min. Some variables showed moderate effect sizes without statistical significance, likely due to limited power. No performance benefits were observed in either condition at 16 min.

**Conclusion:**

Acute BJ intake synergizes with BFR-induced PAP to transiently enhance neuromuscular performance, particularly within an early 8-min window. However, the effects are time-sensitive and may not extend or outperform placebo at later stages. These findings support a targeted, time-optimized use of BJ in warm-up strategies for explosive performance.

## 1 Introduction

Vertical jump performance is a critical indicator of lower limb explosive power in athletes (1). For example, elite basketball players execute 40–60 jumps per game, underlining its importance for success (2). Pre-competition warm-up protocols are therefore essential to optimize neuromuscular function and enhance key athletic capacities such as jumping and agility, while also reducing injury risk (3–9). Consequently, scientifically designed warm-up protocols are indispensable for maximizing athletes’ competitive readiness.

Post-activation potentiation (PAP), characterized by a transient enhancement in neuromuscular performance following prior muscle activation, has become a critical strategy for optimizing warm-up efficacy (10, 11). The extant evidence indicates that near-maximal contractions are requisite for inducing PAP (12). However, practical limitations like equipment dependency and injury risks restrict heavy-load PAP application pre-competition (13). To address this, blood flow restriction (BFR) training has emerged as a promising alternative. BFR utilizes low-load exercises under restricted blood flow, simulating high-intensity stress by promoting metabolite accumulation and triggering neuromuscular adaptations (10, 14, 15). Notably, studies have demonstrated that BFR, especially when combined with plyometric or other exercises, can significantly improve jump performance within 4 to 8 min post-activation (16–18, 55). These findings highlight BFR’s potential to refine pre-competition warm-ups.

Nitric oxide (NO) plays a critical regulatory role in exercise physiology. Dietary nitrate (NO₃^−^) from sources like beetroot juice (BJ) is reduced to nitrite (NO₂^−^) and then to NO, a pathway preferentially enhanced in ischemic/hypoxic conditions—such as those created by BFR (19–21). NO improves skeletal muscle function by modulating calcium handling and blood flow (22–26, 56). The International Olympic Committee recognizes dietary nitrate supplementation as an effective ergogenic aid (27). Beetroot juice (BJ), a rich source of nitrate, has strong evidence for improving performance (28, 29). However, the acute effects of BJ remain debated, with studies reporting improvements in some contexts (30–32) but not others, including in team-sport settings like basketball (33–36).

Despite these mixed findings and the strong theoretical synergy between BJ’s NO-boosting effects and the ischemic environment of BFR, a critical gap exists. Specifically, it remains unexplored whether acute BJ supplementation can synergize with BFR-induced PAP to amplify and potentially accelerate performance gains in basketball-specific, explosive movements. Elucidating this interaction is critical for developing time-optimized, evidence-based warm-up strategies (36). Since BFR creates local tissue ischemia/hypoxia, this is precisely the optimal environment for the NO₃^−^–NO₂^−^–NO pathway. Therefore, this study aims to investigate the acute effects of BJ supplementation combined with BFR-induced PAP on vertical jump performance in male basketball players. By clarifying this potential synergy, this research seeks to inform evidence-based strategies for optimizing pre-competition warm-up protocols.

## 2 Materials and methods

### 2.1 Participants

A total of 20 healthy male basketball players classified as tier 2 or higher were recruited for this study, based on a participant classification framework that defines training and competition level (37).

The exclusion criteria were: History of lower-limb musculoskeletal injuries or surgeries within the past 6 months; Chronic pain or neuromuscular dysfunction; Use of performance-affecting medications/supplements (e.g., creatine, caffeine) within 3 months; Being a smoker or habitual user of mouthwash; Current participation in other sports intervention studies. The participant characteristics are presented in Table 1.

**Table 1:** Participant characteristics.

Sample size	Age (years)	Height (cm)	Body mass (kg)
20	21.25 ± 1.77	177.25 ± 4.79	76.70 ± 6.17

The data are presented as M ± SD.

The study was conducted according to the guidelines of the Declaration of Helsinki and approved by the Sports Science Experiment Ethics Committee of Beijing Sport University (No. 2025134H). Written informed consent was obtained from all study participants.

### 2.2 Experimental design

This study employed a randomized, double-blind, placebo-controlled, counterbalanced crossover design with two supplementation conditions. In the experimental condition, participants consumed nitrate-rich beetroot juice (BJ) (M-ACTION Beetroot Liquid, China). Based on the product’s specification of ≥5 mmol nitrate per 50 mL, a calculated volume was administered to provide a total dose of 8.4 mmol of nitrate. In the placebo condition, participants consumed a visually matched nitrate-depleted juice blend made from nitrate-free natural carotenoids and brown sugar water. In nitrate supplement studies, using a liquid placebo that depletes nitrates but resembles in appearance and flavor is a recognized method to ensure the absence of active components. A 7-day washout period was implemented between the two experimental trials to avoid carryover effects.

Randomization of the condition order (BJ-placebo or placebo-BJ) was achieved using a computer-generated sequence concealed within sealed, opaque envelopes. Double-blinding procedures were strictly implemented: participants remained unaware of the study’s hypothesis and the specific supplement identity, while the researchers responsible for conducting all outcome assessments (warm-up, PAP induction, and performance testing) were kept blinded to the supplementation condition throughout the entire experiment. The researcher who prepared the supplements according to the randomization sequence had no further contact with participants during testing sessions.

### 2.3 Equipment and instrumentation

The color Doppler ultrasound diagnostic instrument utilized in the study was the SIEMENS Cypress PLUS (Siemens Medical Solutions USA, Inc.). Prior to the commencement of the experiment, the participants measured lower extremity arterial occlusion pressure at rest using the device above and a pressurized belt with a manometer. The blood flow restriction belt was procured from Theratools, a company based in China. Based on previous research recommendations, the blood flow restriction band was then positioned over the base of the subject’s thigh, at the level of the transverse gluteal muscle, with a pressure of 50% AOP (13), as part of the induction of PAP. A rigorous data collection process was undertaken, employing KunWei force plates (Kunwei Sports Technology Corporation, China). These plates have been meticulously evaluated for reliability and validity, ensuring the integrity of the collected data (38).

### 2.4 Protocol and control

#### 2.4.1 Measurement of AOP

Two BFR belts were positioned at the proximal thigh, just below the transverse gluteal muscle. Before measurement, the Doppler ultrasound probe was coated with an appropriate coupling agent. To confirm arterial blood flow, the probe is used to detect the dorsalis pedis artery, ensuring the presence of a pulsatile signal or observable waveforms. After 10 minutes of supine rest, pressure had been gradually applied to the BFR belts until the arterial flow signal was fully occluded, followed by a slow, controlled deflation. The pressure at which the arterial signal reappears was recorded as the AOP. This method has been widely adopted in blood flow restriction research and is considered a valid and reliable approach for determining individualized occlusion pressure using Doppler ultrasound (39).

#### 2.4.2 Supplement intake

After randomization, during the 2.5-h rest period after juice consumption, participants were supervised and were not permitted to consume any food, beverages (except water), or use any oral care products such as mouthwash or chewing gum.

#### 2.4.3 Warm-up and baseline measurement

All subjects were familiar with the principles and procedures of CMJ movements. After completing the supplement intake procedure, the subjects warmed up and then underwent a baseline test. The protocol began with a 5-min jog followed by a standardized dynamic stretching routine, which has been shown to effectively prime neuromuscular performance in athletic populations (40). The sequence of movements included the knee–heel lift, goose balance, quadriceps stretch, and maximal stretch, with six repetitions of each movement. Subsequently, the subjects were requested to execute two full-force CMJ on a force platform. The data obtained from these jumps served as the baseline for subsequent analysis.

#### 2.4.4 Induction of PAP

The subjects were fitted with blood flow restriction belts at the base of the thigh and pressurized to 50% AOP. They were then instructed to perform two sets of ten straight-legged jumps, with a 30-s interval and a 90-s completion time. Three sets of five consecutive obstacles followed these jumps, with a height of 50 centimeters, a 30-s interval, and a 120-s completion time. Finally, the subjects performed five drop jumps, with a height of 50 centimeters, a 10-s interval, and a 90-s completion time (41). The entire sequence was completed within a five-minute timeframe. The total duration of these exercises is 5 minutes.

#### 2.4.5 Measurement of PAP effects

**Figure 1:**
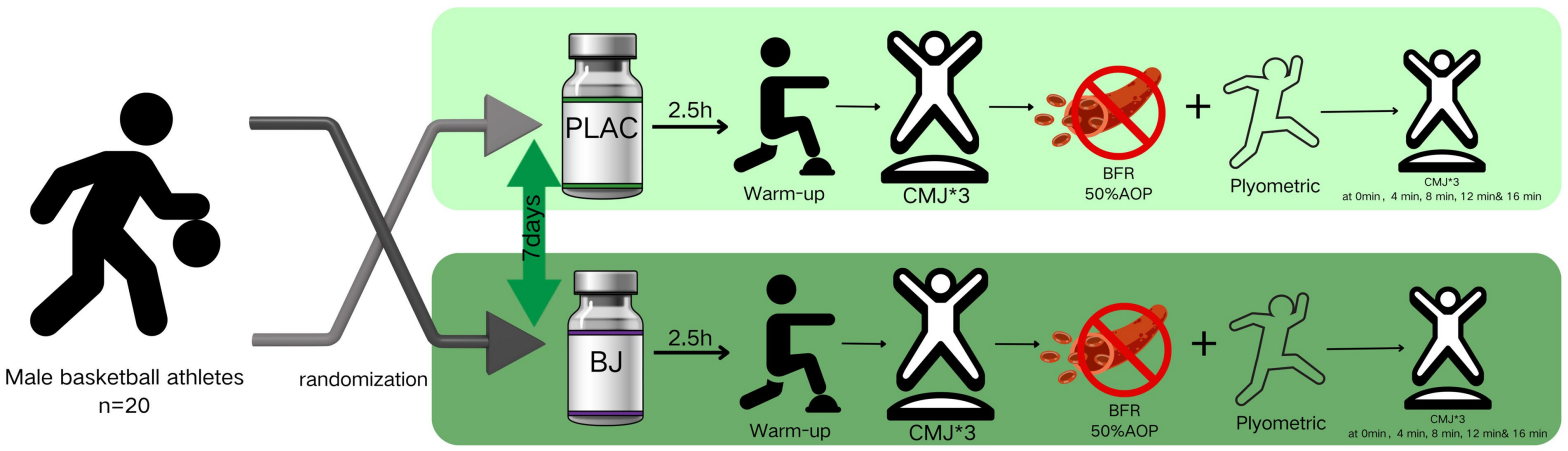
Flowchart of the experiment.

The timing of the subsequent events was initiated subsequent to the subjects’ completion of the plyometric training, and the removal of the blood flow restriction belts. Two CMJ tests were then conducted at 0, 4, 8, 12, and 16 min (42, 43), and the data were recorded. The experimental procedure is illustrated in Figure 1.

### 2.5 Data collection and processing

We collected the jump height calculated based on the ground clearance speed [H(v), m], maximum combined force (MCF) (N), maximum relative force (MRF) (BW), peak power (PP) (w), relative peak power (RPP) (W/kg), peak rate of force development (PRFD, N/s), and reaction force index modified (RSImod). The modified reactive strength index (RSImod) was calculated as jump height (in meters) divided by the time to take-off (in seconds). These metrics are automatically calculated and saved by the KunWei motor function performance test system software(KW3.0.10.9 M, Kunwei, Shanghai, China) (38, 44). Subsequently, the data ought to be imported into Excel in a manual fashion. This will facilitate the organization and analysis of the data. The percentage increase or decrease in each metric at different time points compared to baseline values for each supplement intake condition was calculated.

### 2.6 Statistical analysis

The statistical software SPSS Version 27.0 (IBM Corp., Armonk, NY, USA) was utilized for all statistical analyses. Statistical significance was accepted at *p* < 0.05. The results are presented as mean ± standard deviation (SD). Our study employs a two-factor repeated measures ANOVA to investigate changes in multiple time-point indicators under various conditions. Due to insufficient residual degrees of freedom, multivariate tests are not feasible; therefore, the focus is on reporting univariate test results. Before conducting the repeated measures ANOVA, the Mauchly’s test of sphericity is used to check for sphericity. If the sphericity assumption is not met, the Greenhouse–Geisser correction is applied to adjust the degrees of freedom, and the corrected statistical results are reported to ensure robustness. Paired-sample *t*-tests were conducted to compare the data for each indicator at various time points within a specific supplement intake condition to baseline values. Additionally, paired-sample *t*-tests were used to compare the percentage change (increase or decrease) in the data for each indicator across different time points between the two supplement intake conditions. In addition to reporting the *p* value, the results of paired *T*-test also provide the effect size (Cohen’s d) and 95% confidence interval to reflect the practical significance of the difference. In order to deal with the problem of multiple comparisons, Bonferroni correction was used in the paired *T*-test within the group to balance error control and power retention.

## 3 Result

### 3.1 Effects of plyometric exercise with blood flow restriction on PAP following supplementation with placebo or beetroot juice

The effects of BFR-augmented plyometric exercise on PAP under PL or BJ conditions are summarized in Table 2. Under PL, H(v) significantly increased at 8 min post-exercise versus baseline (*p* = 0.001, *d* = 0.90). PP also rose at 8 min (*p* = 0.007, *d* = 0.67). In contrast, RSImod decreased at 16 min (*p* = 0.006, *d* = 0.69). No other metrics changed significantly after correction. BJ elicited broader PAP effects. H(v) increased immediately post, and at 4 and 8 min (*p* ≤ 0.01, *d* ≥ 0.64). MCF and MRF improved at 4 min (MCF: *p* = 0.008, *d* = 0.67; MRF: *p* = 0.003, *d* = 0.75). PP increased at 0 and 8 min (*p* ≤ 0.004, *d* ≥ 0.74). RPP and PRFD improved from 0 to 8 min and 0 to 4 min, respectively (RPP: *p* ≤ 0.007, *d* ≥ 0.67; PRFD: *p* ≤ 0.006, *d* ≥ 0.68). ANOVA showed a significant main effect of group for H(v), RPP, and RSImod (all *p* = 0.01, η^2^ ≥ 0.30), but not for MCF, MRF, PP, or PRFD. A main effect of time occurred for all variables (*p* ≤ 0.02, η^2^ ≥ 0.17). Significant group × time interactions were found for H(v) (*p* = 0.01, η^2^ = 0.17), PP (*p* = 0.02, η^2^ = 0.15), and RPP (*p* = 0.03, η^2^ = 0.14).

**Table 2:** Effect of inducing PAP in the presence of placebo and beetroot juice intake.

Variables	Time	Placebo			Beetroot juice		
		Mean ± SD	*p*	Cohen’s *d*	Mean ± SD	*p*	Cohen’s *d*
H(v)^G, T, G*T^ (m)	Baseline	0.45 ± 0.06	–	–	0.43 ± 0.07	–	–
	0 min	0.46 ± 0.07	0.355	0.21	0.45 ± 0.08	0.007	0.67
	4 min	0.48 ± 0.07	0.013	0.62	0.44 ± 0.06	0.01	0.64
	8 min	0.48 ± 0.08	0.001	0.90	0.46 ± 0.08	<0.001	1.16
	12 min	0.47 ± 0.07	0.061	0.45	0.43 ± 0.07	0.299	0.24
	16 min	0.46 ± 0.06	0.342	0.22	0.43 ± 0.07	0.76	0.07
MCF^T^ (N)	Baseline	2036.10 ± 176.04	–	–	2048.75 ± 267.26	–	–
	0 min	2023.98 ± 196.28	0.666	0.10	2071.08 ± 270.85	0.352	0.21
	4 min	2109.01 ± 220.22	0.069	0.43	2145.63 ± 358.70	0.008	0.67
	8 min	2039.47 ± 196.47	0.926	0.02	2062.74 ± 245.03	0.74	0.08
	12 min	2042.11 ± 181.50	0.865	0.04	1993.07 ± 255.58	0.078	0.42
	16 min	2033.72 ± 190.08	0.946	0.02	2021.61 ± 242.83	0.427	0.18
MRF^T^ (BW)	Baseline	2.83 ± 0.22	–	–	2.77 ± 0.31	–	–
	0 min	2.84 ± 0.29	0.777	0.06	2.82 ± 0.33	0.141	0.34
	4 min	2.93 ± 0.27	0.057	0.45	2.91 ± 0.44	0.003	0.75
	8 min	2.84 ± 0.22	0.875	0.04	2.80 ± 0.27	0.539	0.14
	12 min	2.85 ± 0.22	0.594	0.12	2.71 ± 0.30	0.155	0.33
	16 min	2.83 ± 0.22	0.959	0.01	2.75 ± 0.28	0.638	0.11
PP^T, G*T^ (W)	Baseline	4542.59 ± 501.64	–	–	4449.19 ± 500.60	–	–
	0 min	4599.61 ± 519.49	0.313	0.23	4583.67 ± 586.88	0.001	0.87
	4 min	4627.42 ± 499.74	0.082	0.41	4496.12 ± 476.51	0.062	0.44
	8 min	4642.54 ± 510.94	0.007	0.67	4582.05 ± 609.52	0.004	0.74
	12 min	4582.57 ± 464.65	0.333	0.22	4395.70 ± 545.88	0.102	0.38
	16 min	4543.98 ± 480.77	0.972	0.01	4384.01 ± 512.90	0.035	0.51
RPP^G, T, G*T^ (W/kg)	Baseline	61.79 ± 6.22	–	–	59.05 ± 6.25	–	–
	0 min	62.63 ± 6.90	0.327	0.23	61.35 ± 7.45	<0.001	1.06
	4 min	63.02 ± 6.42	0.088	0.40	60.07 ± 5.70	0.007	0.67
	8 min	63.29 ± 6.66	0.012	0.62	61.41 ± 7.72	0.001	0.90
	12 min	62.37 ± 5.82	0.324	0.23	58.82 ± 6.78	0.576	0.13
	16 min	61.99 ± 5.96	0.746	0.07	58.65 ± 6.32	0.33	0.22
PRFD^T^ (N/s)	Baseline	13081.47 ± 2908.64	–	–	13055.93 ± 2519.15	–	–
	0 min	12455.37 ± 3002.17	0.288	0.24	14061.30 ± 2394.57	0.002	0.79
	4 min	14686.96 ± 2715.59	0.097	0.39	14599.00 ± 3218.08	0.006	0.68
	8 min	13927.03 ± 2105.14	0.323	0.23	14051.79 ± 2970.25	0.033	0.52
	12 min	13560.72 ± 2583.26	0.606	0.12	12640.48 ± 3195.62	0.366	0.21
	16 min	13971.82 ± 1662.51	0.211	0.29	13117.73 ± 3367.08	0.928	0.02
RSImod^G, T^	Baseline	0.72 ± 0.08	–	–	0.64 ± 0.10	–	–
	0 min	0.71 ± 0.11	0.280	0.25	0.65 ± 0.14	0.457	0.17
	4 min	0.68 ± 0.15	0.115	0.37	0.63 ± 0.14	0.866	0.04
	8 min	0.72 ± 0.12	0.812	0.05	0.63 ± 0.17	0.718	0.08
	12 min	0.68 ± 0.10	0.062	0.44	0.62 ± 0.08	0.065	0.44
	16 min	0.62 ± 0.12	0.006	0.69	0.63 ± 0.07	0.802	0.50

The data are presented as M ± SD. H(v): velocity-calculated jump height. MCF, maximum combined force; MRF, maximum relative force; PP, peak power; RPP, relative peak power; PRFD, peak rate of force development; RSImond, the modified reaction force index. ^G^: The main effect of group is statistically significant. ^T^: The main effect of time is statistically significant. ^G*T^: The main effect of group×time is statistically significant.

### 3.2 Comparative analysis of PAP effects between placebo and beetroot juice intake

Significant between-group differences in percentage change from baseline were observed at two time points. Immediately post-intervention, PRFD was significantly greater in the BJ condition compared to placebo (*p* < 0.05), with a mederate effect size (*d* = 0.62). At 12 min post-intervention, H(v) was significantly higher in the placebo condition (*p* < 0.05), with a moderate effect size (*d* = 0.67). Although other time points did not reach statistical significance (*p* > 0.05), several comparisons showed small-to-moderate effect sizes. For example, RPP exhibited a small effect in favor of BJ at 4 min (*d* = 0.42), and H(v) showed a small effect in favor of BJ immediately post-intervention (*d* = 0.39). These non-significant but non-trivial effect sizes reflect observable between-condition trends.

**Figure 2:**
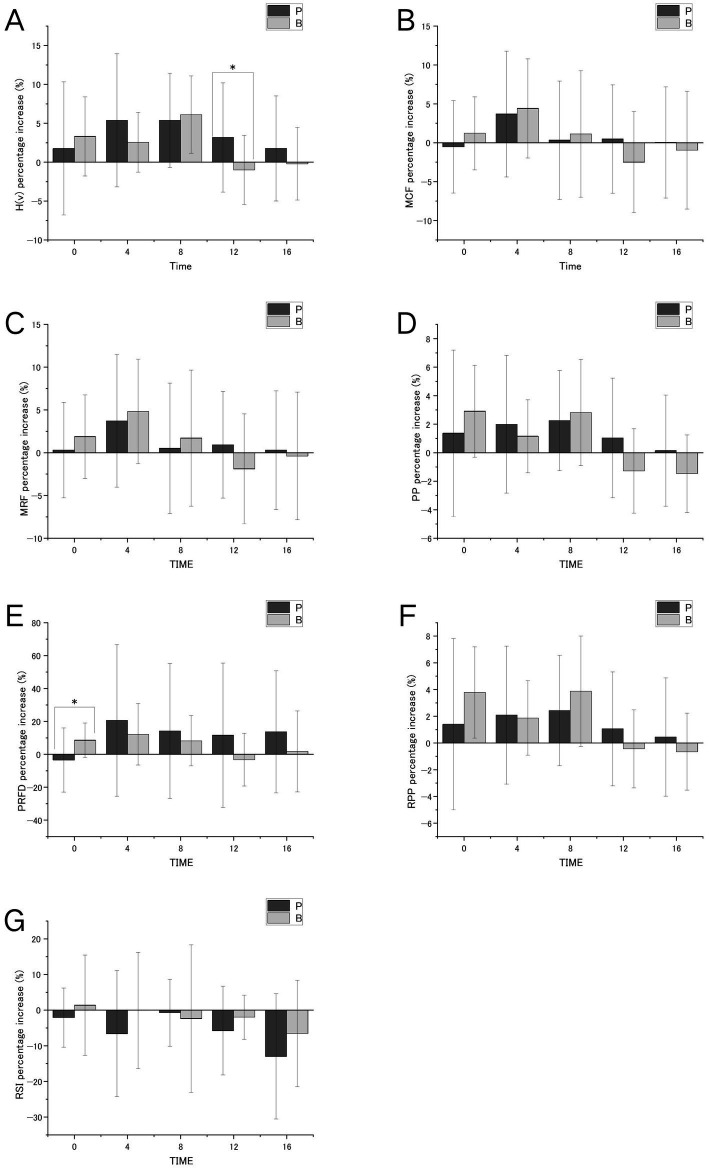
The improvement status of various indicators relative to the baseline at different time points.

Across most outcome variables and time points, between-condition comparisons yielded non-significant *p*-values and small effect sizes (*d* < 0.30), indicating limited differences in percentage change between the two supplementation conditions (see Figure 2).

## 4 Discussion

This study investigated the acute effects of BJ supplementation combined with BFR-induced PAP on vertical jump kinetics in elite male basketball players. The results revealed synergistic yet short-lived interactions between the two interventions, highlighting a critical temporal window for optimizing athletic performance. The results revealed a significant PAP effect under placebo conditions, suggesting that BFR alone may have contributed to the observed performance enhancements. Notably, the placebo condition demonstrated effectiveness at both 4- and 8-min post-activation, followed by a gradual decline. These findings align with previous research indicating that low-load BFR training can enhance PAP by promoting metabolite accumulation and increasing motor unit recruitment (45, 46).

The principal finding of this study is that acute beetroot juice intake synergizes with a blood flow restriction plyometric protocol to transiently enhance key metrics of neuromuscular performance [H(v), PP, PRFD] in elite basketball players, specifically within the critical early 0- to 8-min post-activation window. While the ergogenic effects of BJ and the ability of BFR to induce PAP have been reported independently, this is the first study to demonstrate a time-sensitive synergistic interaction between them. This finding advances our understanding of nutritional and physiological strategies by highlighting that their combined effect is not merely additive but can potentially accelerate the onset of performance enhancement, a crucial factor in pre-competition preparation.

The combined intervention of BJ ingestion and BFR significantly enhanced PAP-induced improvements in H(v), PP, and PRFD during the 0–8 min post-intervention compared to placebo. This enhancement was supported by medium-to-large effect sizes [Cohen’s *d* ≥ 0.64 for H(v), *d* ≥ 0.74 for PP, and *d* ≥ 0.52 for PRFD], indicating clinically meaningful improvements beyond statistical significance. This suggests that BJ may accelerate the onset of PAP, potentially through enhanced nitric oxide bioavailability, which improves muscle oxygenation and calcium handling in the early phase of recovery (22, 24). This environment is precisely the condition under which the non-canonical nitrate-nitrite-NO pathway is most efficient, acting as a potentiation of NO generation from the BJ-derived nitrate pool (19, 20). The elevated NO bioavailability likely enhances early PAP through two primary mechanisms: (1) Improved Calcium Handling: NO may modulate sarcoplasmic reticulum function, increasing the release or sensitivity of calcium (Ca^2+^) ions during excitation-contraction coupling, leading to faster and more forceful contractions (22, 24); (2) Enhanced Perfusion and Metabolic Clearance: The vasodilatory effect of NO, coupled with a potential homogenization of microvascular blood flow upon cuff release (reperfusion), may accelerate the washout of metabolic byproducts (e.g., inorganic phosphate, H^+^ ions) accumulated during the BFR exercise, improving the metabolic milieu for force production (26). This may be attributed to NO precursors in BJ, as increased nitrate and nitrite levels have been shown to rapidly elevate blood flow (34, 47). This increase in blood flow may occur via the nitrate–nitrite pathway, thereby improving muscle oxygenation and calcium regulation during the early phases of PAP. However, further research is needed to elucidate the precise mechanisms and temporal characteristics of this effect.

While no prior studies have examined the acute combination of BJ and BFR on vertical jump performance, our results align with and extend the mechanistic findings of Esen et al. (48). They reported that nitrate supplementation improved motor unit firing rates and contractile function during isometric BFR exercise. Our study demonstrates that this synergy translates to a dynamic, basketball-relevant task and, crucially, identifies a narrow post-activation time window for its practical application.

One notable and unexpected observation was the significantly higher H(v) in the placebo condition at 1.2 min post-intervention. This between-group difference exhibited a moderate effect size (*d* = 0.67), suggesting practical relevance despite the limited sample size. This delayed peak in the placebo group may reflect individual variability in PAP response timing, which has been previously documented (49). This outcome may be explained by several possible mechanisms. First, BJ supplementation could have induced an earlier performance peak due to accelerated NO-mediated vasodilation and muscle oxygenation, as reported by Wylie et al. (50). However, this effect may also have dissipated more quickly due to a mismatch between BJ pharmacokinetics and the timing of optimal PAP expression or due to metabolic fatigue. Second, the placebo group may have experienced a delayed PAP response peaking at 12 min, which aligns with previous reports suggesting inter-individual and protocol-dependent variation in PAP time course (49). Third, the presence of non-responders to dietary nitrate—an established phenomenon in BJ research—could dilute the group-level advantage under BJ, resulting in apparent underperformance relative to placebo despite similar variance (51). The return of PP and RSImod to sub-baseline levels by 16 min post-intervention indicates time-dependent efficacy, revealing a narrow window for performance optimization. The decline in performance after 12 min suggests that the combined effects of BJ and BFR are transient, possibly due to the short half-life of nitric oxide or the onset of metabolic fatigue (35, 52). Notably, no significant enhancements were observed in MCF, MRF, or RSImod during the PAP windows. For MCF and MRF, the observed effect sizes were consistently small (*d* < 0.30), confirming minimal practical impact of either intervention on these measures. This differential effect suggests that the BJ-BFR synergy preferentially benefits metrics related to the rate and explosiveness of force production [H(v), PP, PRFD] rather than maximal absolute force (MCF, MRF). This is consistent with the proposed mechanisms targeting calcium kinetics and metabolic recovery, which are more critical for power output than for maximal strength. A detailed analysis of significance levels and effect sizes across all outcome variables provides a more comprehensive understanding of the observed effects. Under the BJ condition, H(v), PP, and RPP showed statistically significant improvements, all accompanied by medium-to-large partial η^2^ values (≥ 0.30), indicating robust performance enhancement. PRFD did not reach significance (*p* > 0.05) but showed a moderate effect size (partial η^2^ = 0.13), suggesting a possible trend toward improvement. RSImod, although statistically significant over time, had only small-to-moderate effect sizes (≈ 0.19), indicating limited magnitude of change. In contrast, MCF and MRF demonstrated neither statistical significance nor meaningful effect sizes under either condition. This may be explained by two factors: (1) a temporal mismatch between BFR-induced metabolite accumulation—most effective at 4–8 min—and peak NO bioavailability, which typically occurs approximately 2.5 h post-ingestion (35), potentially diminishing synergistic effects; (2) Differential physiological adaptations where NO-mediated microcirculatory and mitochondrial enhancements preferentially benefit endurance activities (31, 53); contrasting with PAP’s reliance on rapid neuromuscular synchronization for explosive power output (35, 52). Collectively, these mechanisms suggest that despite its circulatory advantages, NO may have limited efficacy in supporting high-intensity, power-oriented movements. Moreover, while the ergogenic effects of BJ appear limited in young trained athletes, it is important to recognize that dietary nitrate supplementation may offer greater benefits in populations with lower baseline nitrite availability, such as older adults or clinical populations (54). The observed improvements in H(v) and PP with BJ are consistent with previous findings that nitrate supplementation enhances power output and jump performance in athletes, particularly when combined with ischemic stimuli (48). However, the lack of sustained benefit beyond 8 min underscores the need for precise timing in practical applications.

From a practical standpoint, our findings provide coaches and athletes with a targeted, evidence-based strategy to optimize explosive performance at the start of a game or following halftime. To leverage the synergistic window identified here, we propose the following protocol: athletes should ingest a single dose of BJ (≈8.4 mmol nitrate) approximately 2.5 h before the competition. Subsequently, a BFR-enhanced plyometric protocol (as described in this study) should be scheduled as the final component of the active warm-up, concluding 4 to 8 min before the onset of explosive activity (e.g., the initial jump ball, first offensive set, or a critical defensive sequence). This approach offers a potent, equipment-minimal alternative to traditional heavy-load PAP methods, which are often impractical in a competitive setting.

Several methodological limitations should be acknowledged. First, the sample characteristics limit the generalizability of our findings. The relatively small sample size may have underpowered the detection of effects for some variables, as indicated by moderate effect sizes with low statistical power (e.g., for PRFD, PP, and MRF). Consequently, non-significant outcomes should be interpreted with caution, and future studies with larger samples are warranted. Furthermore, our cohort consisted exclusively of elite male athletes, precluding insights into female populations or recreational athletes. Future research should include female participants, accounting for potential influences of the menstrual cycle. Additionally, the participants were primarily of a similar body stature (e.g., guards), which may limit the applicability of our findings to all basketball player positions (e.g., centers and forwards); thus, investigation in athletes of diverse morphologies is encouraged.

Second, the intervention parameters were standardized rather than individualized. The use of a fixed BFR pressure (50% AOP) and a single nitrate dosage (8.4 mmol) may not represent the optimal stimulus for all athletes. Future dose-response studies are needed to identify individual-specific thresholds for maximizing performance.

Finally, the mechanistic underpinnings of our observations remain unexplored. The physiological basis for the time-dependent effects, such as the delayed decline in RSImod and PP, is not fully clear. Moreover, the absence of biochemical measurements (e.g., plasma nitrate/nitrite levels) means we cannot confirm individual compliance or the precise pharmacokinetic profile of the supplement in our cohort. Integrating concurrent assessments of plasma nitrate/nitrite kinetics, blood lactate, and electromyographic activity in future work could elucidate the metabolic and neuromuscular interactions underlying these patterns. Likewise, longitudinal studies are required to determine whether chronic BJ supplementation can sustain PAP effects or mitigate fatigue during repeated explosive efforts.

From a practical perspective, our findings offer a targeted strategy for optimizing explosive performance at the start of a game. To leverage the identified 0- to 8-min performance window, we propose the following protocol: athletes should ingest a single dose of BJ (≈8.4 mmol nitrate) approximately 2.5 h before competition. A BFR-enhanced plyometric protocol, as described in this study, should then be scheduled as the final component of the active warm-up, concluding 4 to 8 min before tip-off or the initial sprint. This approach provides a potent, equipment-minimal alternative to traditional heavy-load PAP methods, which are often impractical pre-competition.

## 5 Conclusion

This study demonstrates that acute BJ supplementation, when combined with a BFR-based plyometric protocol, enhances countermovement jump performance in elite basketball players, but only within a limited time window. Significant improvements in H(v), PP, and PRFD were observed during the first 8 min post-activation, after which performance returned to baseline or declined. Notably, BJ did not outperform placebo at later time points, and variability in responses highlights the need for individualized strategies. These findings emphasize the importance of precise timing when applying nitrate supplementation in explosive performance contexts and suggest limited benefit beyond the early post-activation period.

## Data Availability

The original contributions presented in the study are included in the article/supplementary material, further inquiries can be directed to the corresponding authors.
